# Evaluation of Prehospital Undertriage in Relation to Trauma Team Activation—Results from a Prospective Study in 12 Level one German Trauma Centers

**DOI:** 10.3390/jcm13061714

**Published:** 2024-03-16

**Authors:** Helena Düsing, Paul Hagebusch, Markus Baacke, Dan Bieler, Michael Caspers, Valentin Clemens, Matthias Fröhlich, Lisa Hackenberg, Renè Hartensuer, Sebastian Imach, Kai Oliver Jensen, Annette Keß, Christian Kleber, Fabian Laue, Rolf Lefering, Mindaugas Maslauskas, Gerrit Matthes, André Nohl, Orkun Özkurtul, Thomas Paffrath, Vera Pedersen, Tristan Pfläging, Kai Sprengel, Philipp Störmann, Heiko Trentzsch, Christian Waydhas, Uwe Schweigkofler, on behalf of the NIS-Trauma TAcTIC Study Group

**Affiliations:** 1 Department of Trauma, Hand and Reconstructive Surgery, University Hospital Muenster, 48149 Muenster, Germany; 2 Department of Trauma Surgery and Orthopaedic Surgery, Berufsgenossenschaftliche Unfallklinik Frankfurt, 60389 Frankfurt am Main, Germany; 3 Department of Trauma Surgery, Hospital of the Merciful Brothers Trier, 54292 Trier, Germany; 4 Department for Trauma Surgery and Orthopedics, Reconstructive Surgery, Hand Surgery, Burn Medicine German Armed Forces Central Hospital Koblenz, 56072 Koblenz, Germany; 5 Department of Orthopaedics and Trauma Surgery, University Hopsital Düsseldorf, Medical School Heinrich Heine University, 40225 Düsseldorf, Germany; 6 Department of Trauma and Orthopedic Surgery, Cologne-Merheim Medical Centre (CMMC), University of Witten/Herdecke, Ostmerheimerstr. 200, 51109 Cologne, Germany; 7 Department of Orthopaedics and Trauma Surgery, Musculoskeletal University Center Munich (MUM), LMU Klinikum, 80336 München, Germany; 8 Center for Trauma Surgery, Orthopaedics, Hand Surgery and Sports Medicine, Klinikum Aschaffenburg-Alzenau, 63739 Aschaffenburg, Germany; 9 Department of Trauma, University Hospital Zurich (USZ), Raemistr. 100, 8091 Zurich, Switzerland; 10 Department of Orthopaedics, Trauma Surgery and Plastic Surgery, Leipzig University Hospital, Liebigstr. 16, 04103 Leipzig, Germany; 11 Department of Trauma and Reconstructive Surgery, Ernst von Bergmann Klinikum Potsdam, 14467 Potsdam, Germany; 12 Institute for Research in Operative Medicine (IFOM), Witten/Herdecke University, 51067 Köln, Germany; 13 Center for Emergency Medicine, BG Klinikum Duisburg, 47249 Duisburg, Germany; 14 Medical Director Rescue Service, Oberhausen Fire Brigade, 46047 Oberhausen, Germany; 15 Department of Trauma and Reconstructive Surgery, BG Klinikum Bergmannstrost, 06112 Halle (Saale), Germany; 16 Department of Trauma and Hand Surgery, Cellitinnen-Severinsklösterchen, Augustinerinnen Hospital, 50678 Köln, Germany; 17 Integrated Emergency Center (INZ), University Medical Center Mannheim UMM, University Hospital Mannheim GmbH, Theodor-Kutzer-Ufer 1-3, 68167 Mannheim, Germany; 18 Hirslanden Clinic St. Anna, Faculty of Health Sciences and Medicine, University of Lucerne, 6006 Lucerne, Switzerland; 19 Department of Trauma and Orthopedic Surgery, University Hospital Frankfurt, 60596 Frankfurt am Main, Germany; 20 Institut für Notfallmedizin und Medizinmanagement (INM), LMU Klinikum, LMU München, 80336 Munich, Germany; 21 Department of Trauma Surgery, University Hospital Essen, University of Duisburg-Essen, 45147 Essen, Germany

**Keywords:** trauma care, trauma systems, undertriage, trauma team activation, field triage

## Abstract

**Background/Objective: **This prospective, multicenter observational cohort study was carried out in 12 trauma centers in Germany and Switzerland. Its purpose was to evaluate the rate of undertriage, as well as potential consequences, and relate these with different Trauma Team Activation Protocols (TTA-Protocols), as this has not been done before in Germany. **Methods**: Each trauma center collected the data during a three-month period between December 2019 and February 2021. All 12 participating hospitals are certified as supra-regional trauma centers. Here, we report a subgroup analysis of undertriaged patients. Those included in the study were all consecutive adult patients (age ≥ 18 years) with acute trauma admitted to the emergency department of one of the participating hospitals by the prehospital emergency medical service (EMS) within 6 h after trauma. The data contained information on age, sex, trauma mechanism, pre- and in-hospital physiology, emergency interventions, emergency surgical interventions, intensive care unit (ICU) stay, and death within 48 h. Trauma team activation (TTA) was initiated by the emergency medical services. This should follow the national guidelines for severe trauma using established field triage criteria. We used various denominators, such as ISS, and criteria for the appropriateness of TTA to evaluate the undertriage in four groups. **Results**: This study included a total of 3754 patients. The average injury severity score was 5.1 points, and 7.0% of cases (*n* = 261) presented with an injury severity score (ISS) of 16+. TTA was initiated for a total of 974 (26%) patients. In group 1, we evaluated how successful the actual practice in the EMS was in identifying patients with ISS 16+. The undertriage rate was 15.3%, but mortality was lower in the undertriage cohort compared to those with a TTA (5% vs. 10%). In group 2, we evaluated the actual practice of EMS in terms of identifying patients meeting the appropriateness of TTA criteria; this showed a higher undertriage rate of 35.9%, but as seen in group 1, the mortality was lower (5.9% vs. 3.3%). In group 3, we showed that, if the EMS were to strictly follow guideline criteria, the rate of undertriage would be even higher (26.2%) regarding ISS 16+. Using the appropriateness of TTA criteria to define the gold standard for TTA (group 4), 764 cases (20.4%) fulfilled at least one condition for retrospective definition of TTA requirement. **Conclusions**: Regarding ISS 16+, the rate of undertriage in actual practice was 15.3%, but those patients did not have a higher mortality.

## 1. Introduction

Trauma is the primary cause of death up to the age of 45 in the United States [1,2]. Trauma care guidelines published by the German Trauma Society (DGU) have considerably reduced the mortality of severely injured patients over the last decade [3]. In the German trauma system, the EMS decide on patients’ allocation to a trauma center, including a trauma team activation (TTA), through an on-scene assessment. The primary goal is to identify severely injured patients and to treat the right patient at the right hospital. This TTA algorithm is subject to ongoing debate. In Germany, so far, TTA is advised if a patient meets the criteria for a high risk for severe injuries (HRSI) or for a moderate risk for severe injuries (MRSI) [4] (Table 1). The guidelines do not lead to different trauma team protocols regarding HRSI and MRSI. Patients not meeting the criteria for HRSI or MRSI are usually still transported to a hospital with surgical coverage.

**Figure 1:**
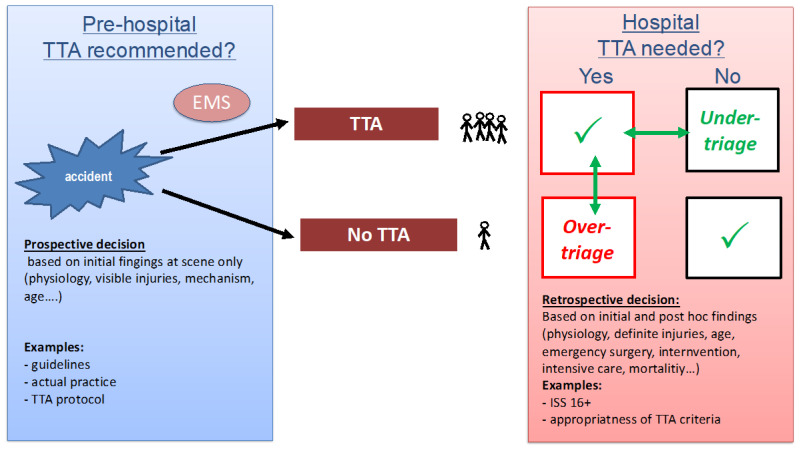
Definition of overtriage and undertriage. Recommended or actual performed prehospital TTA decisions are compared with retrospectively determined criteria for TTA requirement.

The quality of field triage is often evaluated based on the rates of overtriage and undertriage. Overtriage describes the group of patients for whom TTA was initiated but ultimately not required retrospectively. Undertriage, on the other hand, is the portion of severely injured patients who were admitted without TTA. The decision for TTA is made by the EMS after a prehospital assessment of the patients and is therefore a prospective decision. The evaluation of the quality of this decision is made retrospectively based on initial and post hoc findings (Figure 1).

In general, overtriage is considered less critical since full treatment has been applied although less would have been sufficient. Nevertheless, overtriage consumes limited hospital resources. Undertriage is considered to be more critical regarding patient outcome since there would be a delay in severely injured patients receiving their required interventions or they would even receive them too late [5,6,7].

The calculation of undertriage and overtriage requires a “gold standard” that defines the necessity of a trauma team. Frequently, an injury severity score (ISS) of 16+ is used to define these patients [8,9].

However, not every patient with ISS 16+ might need the full trauma team, while several patients with an ISS below 16 might benefit from a TTA [10]. Therefore, in recent years, other criteria, which are sometimes subsumed under the term “need for trauma intervention”, have gained wider acceptance [7,11,12,13,14,15,16,17,18,19] and have been shown to be superior to the classification based on injury severity alone [10,13,17,18]. Examples of these criteria include the transfusion of red blood cells, mechanical ventilation, admission to ICU, or need for interventional radiology [13].

The American College of Surgeons suggests in their trauma guidelines that undertriage should be limited to a maximum of 5% while overtriage should not exceed 50% [7]. There is a wide range [20,21,22,23,24,25] of reported numbers for overtriage and undertriage worldwide, and there are different ways to calculate them.

The purpose of the present investigation is to evaluate the rate of prehospital undertriage in the German trauma system along with its potential causes and potential consequences, and to relate these with different Trauma Team Activation Protocols (TTA-Protocols).

## 2. Methods

### 2.1. Study Design

We conducted a prospective, multicenter observational cohort study in 12 trauma centers in Germany and Switzerland. All 12 participating hospitals are certified as supra-regional trauma centers. Supra-regional trauma centers are the trauma centers with the highest level of care. This study was planned to include at least 3000 trauma admissions to provide a sufficient sample size for subgroup analyses. Based on the expected number of patients per trauma center, a three-month period of data acquisition was planned per hospital.

Each trauma center collected the data during a three-month period in a time window between December 2019 and February 2021. This study adhered to the “Strengthening the Reporting of Observational Studies in Epidemiology (STROBE) statement”, and was performed in accordance with the ethical standards laid down in the Declaration of Helsinki and its modifications. It was approved by the leading ethics committee of the University of Leipzig (reference number 060/18-ek) and, consecutively, by all local ethics committees of the participating institutions. Written informed consent was obtained from the patients as early as possible following injury. There were no specific study interventions, and only routine data were gathered. Here, we report a subgroup analysis for undertriaged patients.

### 2.2. Inclusion Criteria

Included in the study were all consecutive adult patients (age ≥ 18 years) with acute trauma admitted to the emergency department of one of the participating hospitals by the prehospital emergency medical service within 6 h after trauma. The emergency medical service could be staffed with or without an emergency physician.

### 2.3. Data Collection

All data used for this study were available from routine data. The data contained information on age, sex, trauma mechanism, pre- and in-hospital physiology, emergency interventions, emergency surgical interventions, ICU stay, and death within 48 h.

The data were documented pseudonymized in a web-based system hosted at the Institute for Emergency Medicine and Medical Management (INM) of the Ludwig Maximilians University of Munich. Data were checked for plausibility and completeness. The dataset provided to the analysts was completely anonymized.

### 2.4. Trauma Team Activation

Trauma team activation (TTA) was initiated by the emergency medical services after the prehospital assessment of the patient. TTA should follow the national guidelines for severe trauma using established field triage criteria (Table 1) [4]. However, TTA can also be initiated (or obviated) at the discretion of the emergency medical services. The triage criteria are divided into criteria with a high risk for serious injuries (HRSI) and with a moderate risk for severe injuries (MRSI), as described by guidelines valid at the time of the study [4]. All patients estimated to meet none of these criteria were considered to have an unknown risk for severe injuries (URSI).

In accordance with the national trauma guidelines, TTA requires the presence of a basic trauma team that consists of at least three physicians (two surgeons and one anesthesiologist), of whom at least one anesthesiologist and one surgeon must have an attending status, as well as anesthesia and emergency or surgical nurses and radiology personnel. Expanded trauma teams (e.g., visceral surgeons, neurosurgeons, vascular surgeons, and other specialists) must be provided, and need to arrive within 20–30 min after being alerted.

To define the appropriateness of TTA, we used either an ISS 16+ (groups 1 + 3) as a denominator or the fulfillment of at least one criterion to define the need for TTA listed in Table 2 (groups 2 + 4). We decided to use those criteria because they were developed and validated in the German trauma system [8,9].

We used the Cribari matrix (CM) method [17] to calculate overtriage. The CM calculates overtriage by dividing the patients with no need for TTA by all patients receiving TTA. This is equivalent to the false discovery rate, which seems reasonable for the undertriage rate because this focuses on the proportion of unnecessary uses of resources. The primary concern in undertriage is an increased risk of mortality or a worse outcome due to patients receiving no or belated treatment. This is not addressed in the Cribari formula because it would divide the patients with a need for TTA but that did not receive TTA by all patients not receiving TTA; this includes those with no or minor trauma who are not at risk for being undertriaged. This is the reason we used the formula given by Peng et al. [26], because we believe that it describes the undertriage rate better than the Cribari matrix method because it only uses those patients who are at risk of being undertriaged as the denominator (Table 3).

### 2.5. Statistical Analysis

Descriptive analysis included the number of patients and percentages as qualitative data and mean with standard deviation (SD) and median (Md) as continuous data. Continuous variables were compared using the Mann–Whitney U-test, and categorical variables were compared using the Chi-squared test. A *p*-value < 0.05 was regarded as statistically significant. All the analyses were performed with the SPSS statistical software package (version 25, IBM Inc., Armonk, NY, USA).

## 3. Results

### 3.1. The Study Included a Total of 3754 Patients

The average age of the patients was 56.6 years, and 56% were males (*n* = 2097). A penetrating injury mechanism was observed in 190 cases (5%). Falls were the cause of trauma in 57% of cases, and traffic accidents were the second most frequent cause (26%). The average injury severity was 5.1 points, while 7.0% of cases (*n* = 261) presented with ISS 16+. A total of 83 patients (1.0%) died within the first 48 h after admission, and 974 (26%) had a TTA due to prehospital field triage.

### 3.2. Actual Practice/ISS 16+ (Group 1)

Table 4 presents only patients with ISS 16+ to evaluate how successful the actual practice is in the EMS in identifying patients with ISS 16+. The undertriage rate [26] was 15.3%. The undertriaged patients were remarkably older compared to those treated with a TTA (median 80 vs. 55 years). None of the undertriaged patients showed a systolic blood pressure under 90 mmHg. Almost half of the undertriaged patients showed an isolated brain injury (42.5%). The median ISS in this group was lower (17 vs. 22) and intensive care was needed only half as often as in the TTA group (42.5% vs. 83.3%). Two patients (5%) died within 48 h in the undertriaged group compared to 10% in the TTA group. Both non-survivors in the no-TTA group had an isolated head injury (AIS 5), ASA 3, GCS scores of 3 and 10, and were aged 90 and 95 years.

Of the patients with TTA, 74.2% showed an HRSI and 20% were not treated with TTA even though they presented with an HRSI (Table 4).

### 3.3. Actual Practice/Appropriateness of Trauma Team Activation (Group 2)

If the criteria for the appropriateness of trauma team activation [11,12] were used (*n* = 764) as the gold standard to define which patients need a TTA (Table 5), TTA only occurred in 490 cases (64.1%); thus, the proportion of patients without TTA was 35.9% (*n* = 274), and therefore the undertriage rate was much higher. Patients who were undertriaged according to the appropriateness of TTA criteria were considerably older (median 74 versus 54 years) but were less seriously injured (median ISS 4 versus 13). Forty-eight cases without TTA (17.5%) presented with at least one HRSI criterion. Nine patients died in that group; their median age was 84 years (range 73–95), and none of them required emergency surgery. The median days in the ICU was 3 in both groups. More patients (5.9%) died within 48 h compared to the no-TTA group (3.3%) (Table 5).

### 3.4. Hypothetical Triage According Guideline Criteria/ISS 16+ (Group 3)

According to the S3 guidelines, 525 patients (14.0%) fulfilled at least one HRSI criterion. Another 458 cases fulfilled no HRSI criteria but did meet an MRSI criterion. Thus, according to the guidelines, 983 patients (26.2%) should have had a TTA.

Descriptive data of patients with and without recommendation for TTA are given in Table 6. Patients with ISS 16+ (23%) who were undertriaged according to the formula given by Peng et al. [26] frequently presented with an isolated head injury (35%) or extremity injuries (47%), and 70% required intensive care. The 60 patients who did not receive a TTA did not show a higher mortality (5.0%) than those with TTA (11.4%), even though admission to ICU was high in the no-TTA group (70%) (Table 6).

### 3.5. Guideline Recommendation/Appropriateness of TTA Criteria (Group 4)

In total, 764 cases (20.4%) fulfilled at least one condition for the retrospective definition of TTA requirement when using the appropriateness of TTA criteria as the gold standard for defining TTA. The need for TTA was considered about three times more frequently than according to the ISS 16+ definition. All but 17 cases with ISS 16+ (93.5%) were defined as requiring TTA based on the appropriateness of TTA criteria. A TTA occurred in 437 cases (57.2%); therefore, the undertriage rate would be 42.8% (*n* = 327) based on the appropriateness of TTA criteria.

The undertriage group is particularly characterized by elderly patients (median age 70 versus 55 years) with need for intensive care (44%). The median ISS was low (5 versus 13), but nine patients died (Table 7).

## 4. Discussion

The main goal is to treat all patients adequately. This leads to the need for adequate criteria for TTA. However, prehospital TTA-Protocols may not be able to identify all patients in need of TTA, which denies TTA to these cases (undertriage). Moreover, these protocols can also result in the trauma team being activated for patients without the need for it (overtriage) [7].

Overtriage is extensive [18,19,20] and consumes finite resources in cases of mass casualties and could therefore contribute to a negative overall survival rate [10].

However, undertriage is known to be associated with higher mortality [8,21] and accordingly is a patient hazard. It appears obvious that the definite consequence of undertriage depends on the trauma care system. We could not show any negative effect of undertriage in terms of mortality, ICU admission, or ICU stay in our cohort, even though the rates of undertriage were 15.3% in terms of actual practice and ISS 16+. The reason might be the fully developed German trauma system, including the fact that our cohort was only treated in supra-regional trauma centers and by the same medical team regardless of whether they had a TTA or not. We do not know if the outcomes of these patients could have been even better with TTA. Trauma systems vary broadly, not only among high-, middle-, and low-income countries, but even within Europe [22,23,24,25,26]. Undertriage in different trauma systems and especially in low-to-middle-income countries might make a big difference for the patient in terms of neurosurgical treatment because even patients with moderate traumatic brain injury might benefit from structured neurotrauma service, and task-sharing in neurosurgery is not raising concerns in these countries [27,28].

So, undertriage might also lead to worse outcome depending on the system. According to The American College of Surgeons, undertriage is defined as severely injured patients transported to lower-level trauma centers or other acute care facilities [7]. For those reasons, undertriage is considered to be limited to a maximum of 5% while overtriage should not exceed 50% according to the ACS recommendations [9]. However, as explained in the Methods section, there is more than one way to calculate undertriage. We used the formula described by Peng et al. [26]; this must be taken into account when comparing undertriage rates.

Isolated brain injuries in elderly patients showed a higher mortality rate, but we do not know whether patients did not receive a full TTA due to declared limitations of treatment or because the severity of brain injury was not detected in the prehospital setting.

We used the duration of intensive care, emergency surgeries, and mortality as indirect assessments of adequate initial therapy, as is common in major trauma care.

The German S3 guidelines implemented the HRSI and MRSI criteria in order to identify severely injured patients and lower the rate of over- and undertriage; however, a clear additional burden on the healthcare system was observed, as explained by Marzi et al. [29]. Some of the HRSI and MRSI criteria were eliminated from the new version of the guidelines [30] after a literature review carried out with the aim of generating updates to the guidelines.

Our work showed that if the guidelines were to be strictly followed, we would see an even higher undertriage rate (23% vs. 15%). Actual practice seems to work better, as it provides a lower undertriage rate. Further studies might show which triage criteria might show a lower undertriage rate as well as a moderate overtriage rate in order to achieve the goal of treating all patients adequately and to take the limited resources of TTA into account.

We also used different ways to define over- and undertriage in our study. In order to evaluate the actual practice and the S3 guidelines, we used the most common ISS of 16+ to define who needed a TTA as well as the appropriateness of TTA criteria.

We used ISS 16+ to define patients needing TTA, even though earlier work showed this might not be the best criterion [10]. Waydhas et al. showed that other criteria, such as life-saving procedures, might be better for a post hoc definition of which patients need TTA. These criteria are methodologically well developed, but the current guideline recommendations are not able to select those patients, and if they are used to define who needs a TTA, we would see three times more patients with a need for TTA. So, these criteria might need further studies and a transfer to prehospital parameters might be needed. The fact that the quality of field triage is mostly measured by post hoc criteria shows how difficult it is to provide good prehospital algorithms. A machine learning-based triage algorithm, such as that invented by Senda et al. for evaluating the entry of trauma patients into hybrid operating theaters [31], might be needed for TTA as well.

In group 4, we evaluated whether the HRSI + MRSI criteria can identify patients meeting the appropriateness of TTA criteria. The high rate of undertriaged patients shows that this is not working, but those patients might not need a TTA when considering the low severity of the injuries in the no-TTA group.

The appropriateness of TTA criteria are methodologically well developed but might need more validation and review to demonstrate their suitability; it might even be necessary to meet two criteria.

The fact that patients who were undertriaged in the actual practice group were much older and had mostly isolated head injuries leads to the conclusion that this group might need further attention to lower the undertriage rate in this vulnerable group.

In summary, an update to the current S3 guidelines is necessary, with special attention to older patients; this update was made in the 2022 version of the guidelines. In future investigations, we might see a positive effect of this update on lowering over- and undertriage.

## 5. Limitations

A limitation of this study is the fact that only supra-regional centers participated; for this reason, it is not possible to make a general statement regarding the undertriage rate in the German trauma system. The HRSI and MRSI are only recommendations, and we cannot know for certain why the EMS decided on TTA because this was not documented. Most of the criteria used to evaluate undertriage are post hoc criteria and cannot be used in a prehospital setting by the EMS to make a decision on whether a TTA is needed. Our results regarding the negative effect of undertriage might not be transferable to smaller trauma centers and other trauma systems.

## 6. Simplified Summary

In summary, undertriage based on HRSI or MRSI criteria did not lead to a worse outcome in our small study cohort in terms of ICU stay and mortality within 48 h. But this cannot be transferred to different trauma systems. However, we did not find any severe negative effects due to undertriage after retrospectively applying the appropriateness of TTA criteria as published by Waydhas and Bieler et al. [12] or using ISS 16+. Therefore, the actual criteria used for TTA activation need to be refined to not only reduce overtriage rates but also prevent as much undertriage as possible. Remarkably, undertriaged patients in the actual practice groups (1 + 2) were up to 25 years older (Md 55 vs. 80). This might provide an important consideration when developing new valid criteria for TTA activation.

## Figures and Tables

**Table 1:** Criteria for the prehospital triage for trauma team activation [4].

High Risk for Severe Injuries
Vital Signs
Systolic blood pressure below 90 mmHg
GCS < 9
Respiratory disturbance/intubation requirement
Obvious Injuries
Penetrating injuries of the trunk/neck region
Gunshot injuries of the trunk/neck region
Fractures of more than two proximal bones
Unstable thorax
Unstable pelvic fracture
Amputation injury proximal to hands/feet
Injuries with neurological paraplegic symptoms
Open cranial injuries
Burn > 20% of grade > 2b
Moderate Risk for Severe Injuries
Trauma Mechanism
Fall from a height of more than three meters
Traffic accident
Frontal impact with intrusion of more than 50–75 cm
Speed changes of delta > 30 km/h
Pedestrian/two-wheeler collision
Death of an occupant
Ejection of an occupant

**Table 2:** List of criteria to define the need of trauma team activation [11,12].

Injury Severity
Maximum Abbreviated Injury Scale (AIS) severity ≥ 4
Intensive care (without intermediate care)
ICU stay > 24 h
Mortality
Death within 24 h
Invasive measures (prehospital or in the trauma room)
Cardio-pulmonary resuscitation
Advanced airway management
Chest tube or needle decompression
Pericardiocentesis
Application of a tourniquet (prehospital)
Administration of catecholamines
Blood transfusion
Surgical/radiological therapeutic intervention (before ICU admission)
Life-saving/organ-saving/extremity-saving surgery
Radiological therapeutic intervention
≥2 external fixators (humerus, femur, pelvis)
Impaired vital functions (prehospital or on admission)
Pulse oximetry (SpO_2_) < 90%
Respiratory rate < 9 or >29/min
Systolic blood pressure < 90 mmHg
Shock index > 0.9
Glasgow Coma Scale (GCS) < 9
Drop in GCS of 2 or more points prior to admission
Hypothermia < 35 °C

**Table 3:** Formulas to calculate undertriage following Peng et al. [26] and overtriage using the Cribari matrix method [13,17,18].

	Minor Trauma	Major Trauma	Total
Full trauma team activation	a	b	a + b
Limited or no trauma team activation	c	d	c + d
Total	a + c	b + d	N

Undertriage rate = $\frac{d}{b+d}$; overtriage rate = $\frac{a}{a+b}$ (Cribari matrix method).

**Table 4:** Patients with injury severity score ≥ 16 (ISS 16+) with respect to trauma team activation.

	TTA *n* = 221 (84.7%)	No TTA *n* = 40 (15.3%)	*p*-Value
Age (years, SD)	53.7 (21.8) Md 55	71.9 (18.1) Md 80	<0.001
Male	163 (73.8%)	24 (60.0%)	0.076
Glasgow Coma Scale ≤ 8 *	62 (28.1%)	1 (2.5%)	<0.001
Systolic blood pressure < 90	34 (15.4%)	0 (0%)	0.008
High risk for severe injuries	164 (74.2%)	8 (20.0%)	<0.001
Abdominal injury	45 (20.4%)	2 (5.0%)	0.020
Spine injury	89 (40.3%)	4 (10.0%)	<0.001
Extremity injury	116 (52.5%)	17 (42.5%)	0.245
Isolated head injury	35 (15.8%)	17 (42.5%)	<0.001
ISS	24.6 (8.9) Md 22	18.4 (3.4) Md 17	<0.001
ISS 16+	100%	100%	---
Emergency surgery	73 (33.0%)	3 (7.5%)	0.001
Admission to ICU	184 (83.3%)	17 (42.5%)	<0.001
Days in ICU	8.2 (9.8) Md 4	6.4 (7.8) Md 3	0.309
Death (within 48 h)	24 (10.0%)	2 (5.0%)	0.255

SD: standard deviation; Md: median; ICU: intensive care unit; * prehospital or on admission.

**Table 5:** Patients with trauma team activation requirement according to the list of criteria for the appropriateness of trauma team activation (see Table 2).

	TTA *n* = 490 (64.1%)	No TTA *n* = 274 (35.9%)	*p*-Value
Age (years, SD)	53.3 (22.1) Md 54	64.3 (25.4) Md 74	<0.001
Male	343 (70.0%)	133 (48.5%)	<0.001
Glasgow Coma Scale ≤ 8 *	101 (20.6%)	9 (3.3%)	<0.001
Systolic blood pressure < 90	51 (10.4%)	23 (8.4%)	0.367
High risk for severe injuries	309 (63.1%)	48 (17.5%)	<0.001
Abdominal injury	65 (13.3%)	4 (1.5%)	<0.001
Spine injury	160 (32.7%)	26 (9.5%)	<0.001
Extremity injury	224 (45.7%)	142 (51.8%)	0.105
Isolated head injury	76 (15.5%)	30 (10.9%)	0.080
Injury severity score (ISS)	14.8 (11.1) Md 13	6.3 (5.8) Md 4	<0.001
ISS 16+	212 (43.3%)	32 (11.7%)	<0.001
Emergency surgery	122 (24.9%)	18 (6.6%)	<0.001
Admission to ICU	384 (78.4%)	76 (27.7%)	<0.001
Days in ICU	6.7 (8.5) Md 3	5.1 (5.5) Md 3	0.841
Death (within 48 h)	29 (5.9%)	9 (3.3%)	0.108

Md = median; * prehospital or on admission.

**Table 6:** Patients with ISS 16+ (*n* = 261), grouped according to the guideline recommendation for TTA (at least one high risk for severe injuries or moderate risk for severe injuries criterion present).

	TTA Recommended According to Guideline *n* = 201 (77.0%)	TTA Not Recommended *n* = 60 (23.0%)	*p*-Value
Age (years, SD)	53.8 (24.9) Md 55	65.4 (22.5) Md 60	<0.001
Male	143 (71.1%)	44 (73.3%)	0.741
Glasgow Coma Scale ≤ 8 *	58 (28.9%)	5 (8.3%)	0.001
Systolic blood pressure < 90	34 (16.9%)	0 (0%)	<0.001
High or moderate risk for severe injuries	100%	0%	---
Abdominal injury	41 (20.4%)	6 (10.0%)	0.066
Spine injury	84 (41.8%)	9 (15.0%)	<0.001
Extremity injury	105 (52.2%)	28 (46.7%)	0.449
Isolated head injury	31 (15.4%)	21 (35.0%)	<0.001
Injury severity score (ISS)	24.9 (9.1) Md 22	19.5 (4.5) Md 18	<0.001
ISS 16+	100%	100%	---
Emergency surgery	68 (33.8%)	8 (13.3%)	0.002
Admission to ICU	159 (79.1%)	42 (70.0%)	0.141
Days in ICU	6.7 (8.5) Md 3	5.1 (5.5) Md 3	0.156
Death (within 48 h)	23 (11.4%)	3 (5.0%)	0.144

Md = median; * prehospital or on admission.

**Table 7:** Patients with TTA requirement according to the appropriateness of TTA criteria (*n* = 764), grouped according to the guideline recommendation for TTA (at least one high risk for severe injuries or moderate risk for severe injuries criterion present).

Appropriateness of TTA Criteria	TTA Recommended According to Guideline *n* = 437 (57.2%)	TTA Not Recommended *n* = 327 (42.8%)	*p*-Value
Age (years, SD)	54.3 (22.2) Md 55	61.3 (25.4) Md 70	<0.001
Male	299 (68.4%)	177 (54.1%)	<0.001
Glasgow Coma Scale ≤ 8 *	102 (23.3%)	8 (2.4%)	<0.001
Systolic blood pressure < 90	70 (16.0%)	4 (1.2%)	<0.001
High or moderate risk for severe injuries	100%	0%	---
Abdominal injury	58 (13.3%)	11 (3.4%)	<0.001
Spine injury	138 (31.6%)	48 (14.7%)	<0.001
Extremity injury	210 (48.1%)	156 (47.7%)	0.924
Isolated head injury	61 (14.0%)	45 (13.8%)	0.938
Injury severity score (ISS)	14.9 (11.4) Md 13	7.4 (6.7) Md 5	<0.001
ISS 16+	191 (43.7%)	53 (16.2%)	<0.001
Emergency surgery	110 (25.2%)	30 (9.2%)	<0.001
Admission to ICU	316 (72.3%)	144 (44.0%)	<0.001
Days in ICU	6.4 (8.5) Md 3	4.6 (5.9) Md 2	0.236
Death (within 48 h)	29 (6.6%)	9 (2.8%)	0.015

Md = median; * prehospital or on admission.

## Data Availability

The dataset generated and analyzed during the current study is not publicly accessible but is available from the corresponding author on reasonable request.
